# Genome-wide comparative analysis of putative Pth11-related G protein-coupled receptors in fungi belonging to Pezizomycotina

**DOI:** 10.1186/s12866-017-1076-5

**Published:** 2017-07-25

**Authors:** Xihui Xu, Guopeng Li, Lu Li, Zhenzhu Su, Chen Chen

**Affiliations:** 1 College of Life Sciences, Nanjing Agricultural University, Nanjing, 210095 China; 2 Agricultural Product Processing Research Institute, Chinese Academy of Tropical Agricultural Sciences, Zhanjiang, 524001 China; 3 State Key Laboratory of Rice Biology, Institute of Biotechnology, Zhejiang University, Hangzhou, 310058 China

**Keywords:** Fungi, G-protein coupled receptors, Gene family evolution, Gene expression pattern, Pezizomycotina, Phytopathogens, Phylogenetics analysis, Pth11-related GPCRs

## Abstract

**Background:**

G-protein coupled receptors (GPCRs) are the largest family of transmembrane receptors in fungi, where they play important roles in signal transduction. Among them, the Pth11-related GPCRs form a large and divergent protein family, and are only found in fungi in Pezizomycotina. However, the evolutionary process and potential functions of Pth11-related GPCRs remain largely unknown.

**Results:**

Twenty genomes of fungi in Pezizomycotina covering different nutritional strategies were mined for putative Pth11-related GPCRs. Phytopathogens encode much more putative Pth11-related GPCRs than symbionts, saprophytes, or entomopathogens. Based on the phylogenetic tree, these GPCRs can be divided into nine clades, with each clade containing fungi in different taxonomic orders. Instead of fungi from the same order, those fungi with similar nutritional strategies were inclined to share orthologs of putative Pth11-related GPCRs. Most of the CFEM domain-containing Pth11-related GPCRs, which were only included in two clades, were detected in phytopathogens. Furthermore, many putative Pth11-related GPCR genes of phytopathogens were upregulated during invasive plant infection, but downregulated under biotic stress. The expressions of putative Pth11-related GPCR genes of saprophytes and entomopathogens could be affected by nutrient conditions, especially the carbon source. The gene expressions revealed that Pth11-related GPCRs could respond to biotic/abiotic stress and invasive plant infection with different expression patterns.

**Conclusion:**

Our results indicated that the Pth11-related GPCRs existed before the diversification of Pezizomycotina and have been gained and/or lost several times during the evolutionary process. Tandem duplications and trophic variations have been important factors in this evolution.

**Electronic supplementary material:**

The online version of this article (doi:10.1186/s12866-017-1076-5) contains supplementary material, which is available to authorized users.

## Background

Fungi live in a complex environment, where they receive and integrate abiotic and biotic stimuli, then respond in the manner most appropriate for survival. For example, fungal endophytes in the rhizosphere recognize and colonize specific host plants from which they obtain nutrients [1, 2]. However, the cell wall and membrane, acting as a barrier, separate the interior of the cell from the outside environment [3, 4]. Consequently, communication of cells, both with their environment and with each other, is crucial for the survival of fungi.

Membrane proteins play several essential roles in a cell, including receiving extracellular signals and triggering intracellular responses to them, and the maintenance of interactions between cells [5–7]. The fungal cell membrane is equipped with many protein receptors. These receptors sense both abiotic and biotic stimuli from the surrounding environment, and facilitate the response to these stimuli, which may include altering fungal development, morphogenesis, and metabolism [8, 9]. Cell-surface G-protein coupled receptors (GPCRs) are the largest family of transmembrane receptors, and are characterized by seven transmembrane domains anchored in the plasma membrane with an intracellular carboxyl- and extracellular amino-terminus [10, 11]. GPCRs sense a diverse array of stimuli including light, sugars, amino acids, and pheromones [12, 13]. In fungi, many signaling pathways are regulated by GPCRs, such as the mitogen-activated protein kinase and cAMP-dependent protein kinase cascades. These pathways regulate growth, morphogenesis, metabolism, mating, virulence, and stress responses [14, 15].

Many GPCR receptors have been identified in fungi, including pheromone receptors, cAMP receptor-like receptors, carbon-sensing receptors, Stm1-related receptors, and regulator of G protein signaling (RGS) proteins [16]. After first being identified in *Saccharomyces cerevisiae* [17], Ste2- and Ste3-like pheromone receptors have been found in many ascomycete fungi, while basidiomycete pheromone receptors are only of the Ste3-like type [16, 18]. *Neurospora crassa* GPR-1 was the first cAMP receptor-like GPCR characterized in ascomycete fungi [19] and the number of this type of GPCR varies among fungal species [16]. *S. cerevisiae* Gpr1p and *N. crassa* GPR-4 are carbon-sensing receptors [20, 21], and homologues of Gpr1p and GPR-4 are universally present in fungi [16]. *S. pombe* Stm1 is involved in the recognition of nitrogen starvation signals [22], and Stm1-related receptors are widely distributed in fungi [16]. RGS proteins are GTPase-activating proteins, which provide negative control of Gα protein signaling [23]. GprK were found to contain an RGS domain in *Aspergillus* sp. [24], and GprK homologues are present in ascomycetes [16].

A novel class of receptors, the Pth11-related group, was identified by Kulkarni et al. [25]. This group is typified by *Magnaporthe oryzae* Pth11, a cell-surface integral membrane protein implicated in pathogenesis [25, 26]. These Pth11-related proteins share many characteristics diagnostic of GPCRs, including seven transmembrane regions. For Pth11-related GPCRs, conserved residues (termed as Pth11-domain) occur within the membrane-spanning regions, which is consistent with other GPCRs that sequence conservation is typically limited to the transmembrane sequences [25]. It has been showed that the Pth11-domain is remarkably different from the conserved sequences of other GPCR classes, such as domains conserved in cAMP-, STM1-, and mPR-related GPCRs [25]. The conserved Pth11-domain distinguishes Pth11-related proteins from others and defines a new class of GPCR-like proteins. Except for Pth11-domain, Pth11 also has an amino-terminal extracellular cysteine-rich CFEM domain (pfam05730). However, only a subset of Pth11-related proteins from *M. grisea* and *N. crassa* contained the CFEM domain, and these CFEM domain-containing proteins occur together in one clade on the phylogeny tree [25], indicating that the sequences are closely related. The gene duplication may leads to the arisen of these CFEM domain-containing proteins [25].

The Pth11-related GPCRs form a large and diverse protein family [16, 25]. Interestingly, Pth11-related GPCRs were only found in fungi belonging to Pezizomycotina (a subphylum within Ascomycota), while none were found in other subphyla of Ascomycota or Basidiomycota [16, 25]. These results reveal that Pth11-related GPCRs are very ancient in origin, and may have evolved to serve functions specific to this subphylum of fungi. However, the previous studies only focused on some phytopathogens such as *M. oryzae* and *Fusarium graminearum* while few of them covered symbionts or entomopathogens. Besides, the evolution of Pth11-related GPCRs and their potential functions are largely unknown, especially at the subphylum level. Recently, an increasing number of genome sequences have become available for fungi in Pezizomycotina, making it possible to mine and compare Pth11-related GPCR sequences. Here we explore the genomes of 20 model organisms in Pezizomycotina with different nutritional strategies and identify Pth11-related GPCRs in the deduced proteomes. The phylogenetic analysis and chromosomal distribution has shed light on the evolution of Pth11-related GPCRs. We also mined expression trends for Pth11-related GPCR genes during growth and invasion, as well as under biotic and abiotic stress, using existing mRNA profiles or microarray datasets.

## Results

### Identification of putative Pth11-related GPCRs in Pezizomycotina

**Fig. 1:**
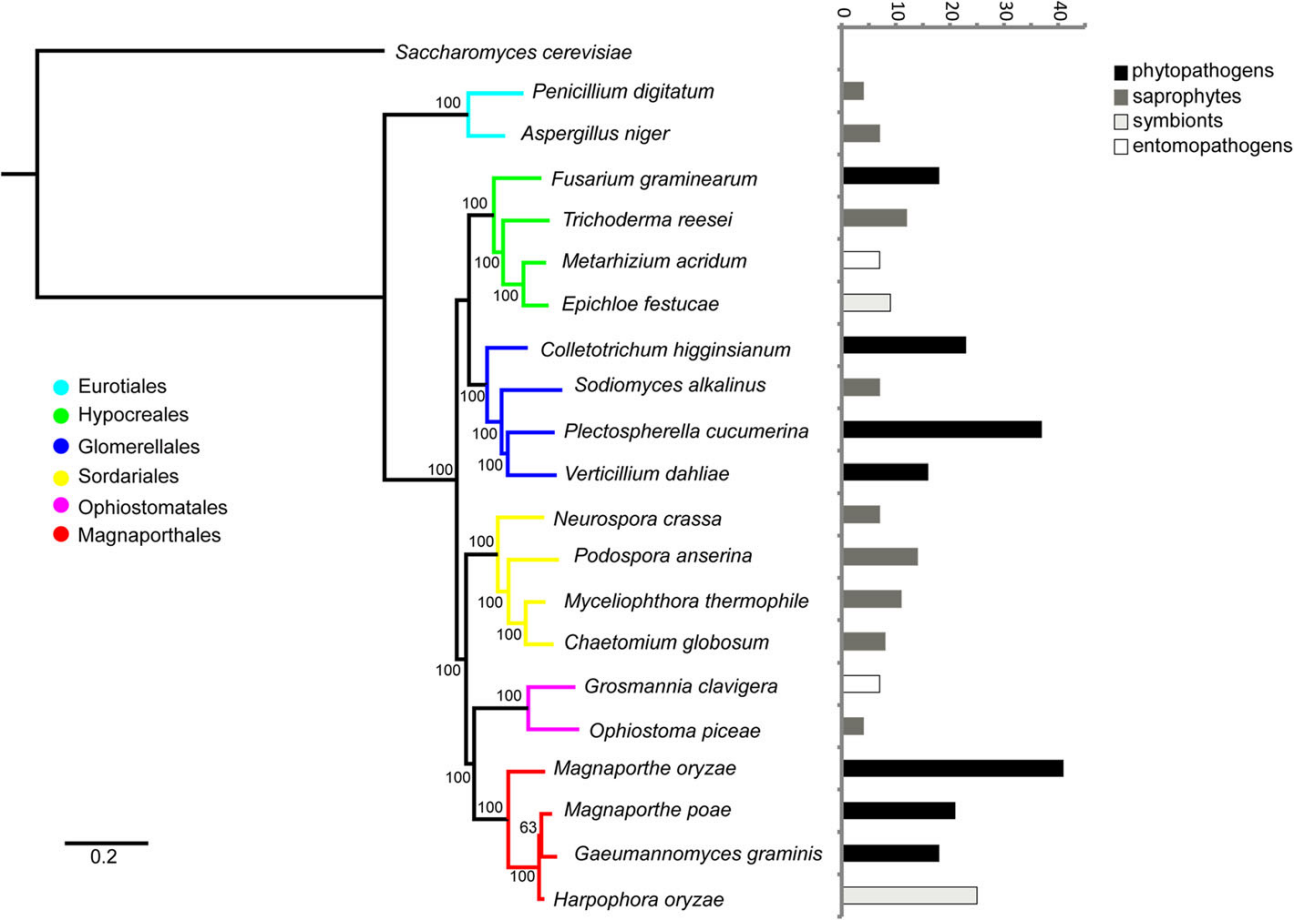
Phylogenetic relationships and number of putative Pth11-related GPCRs among 20 selected fungal species. *S. cerevisiae* was used as outgroup. Maximum likelihood (ML) phylogenetic tree shows the evolutionary relationships of 2 Eurotiales species (cyan), 4 Hypocreales species (green), 4 Glomerellales species (blue), 4 Sordariales species (yellow), 2 Ophiostomatales species (purple), and 4 Magnaporthales species (red). The ML bootstrap values are sequentially indicated above the branches. Numbers of Pth11-related GPCRs of the same 20 fungal species are shown on the right of the phylogenetic tree, including 7 phytopathogens, 9 saprophytes, 2 entomopathogens, and 2 symbionts

In total, 20 genomes of fungi in Pezizomycotina were searched for putative Pth11-related GPCRs using a homology (hmmscan and BLAST)-based strategy. These species include members of the Magnaporthales, Ophiostomatales, Sordariales, Glomerellales, Hypocreales, and Eurotiales (Fig. 1), and cover phytopathogens (*M. oryzae*, *Gaeumannomyces graminis*, *Magnaporthe poae*, *Verticillium dahlia*, *Colletotrichum higginsianum*, *F. graminearum*, and *Plectospherella cucumerina*), symbionts (*Harpophora oryzae*, and *Epichloe festucae*), saprophytes (*Ophiostoma piceae*, *N. crassa*, *Chaetomium globosum*, *Myceliophthora thermophile*, *Podospora anserina*, *Sodiomyces alkalinus*, *Trichoderma reesei*, *Aspergillus niger*, and *Penicillium digitatum*), and entomopathogens (*Grosmannia clavigera* and *Metarhizium acridum*) (Fig. 1). All the identified proteins were evaluated for the typical topology of seven transmembrane regions, which resulted in 296 putative Pth11-related GPCRs being identified in the 20 proteomes (Fig. 1). The different numbers of predicted Pth11-related GPCRs between the present research and previous studies may result from the newer genome database version and strengthened hmmscan and BLAST cut-off to avoid the false positive.

**Fig. 2:**
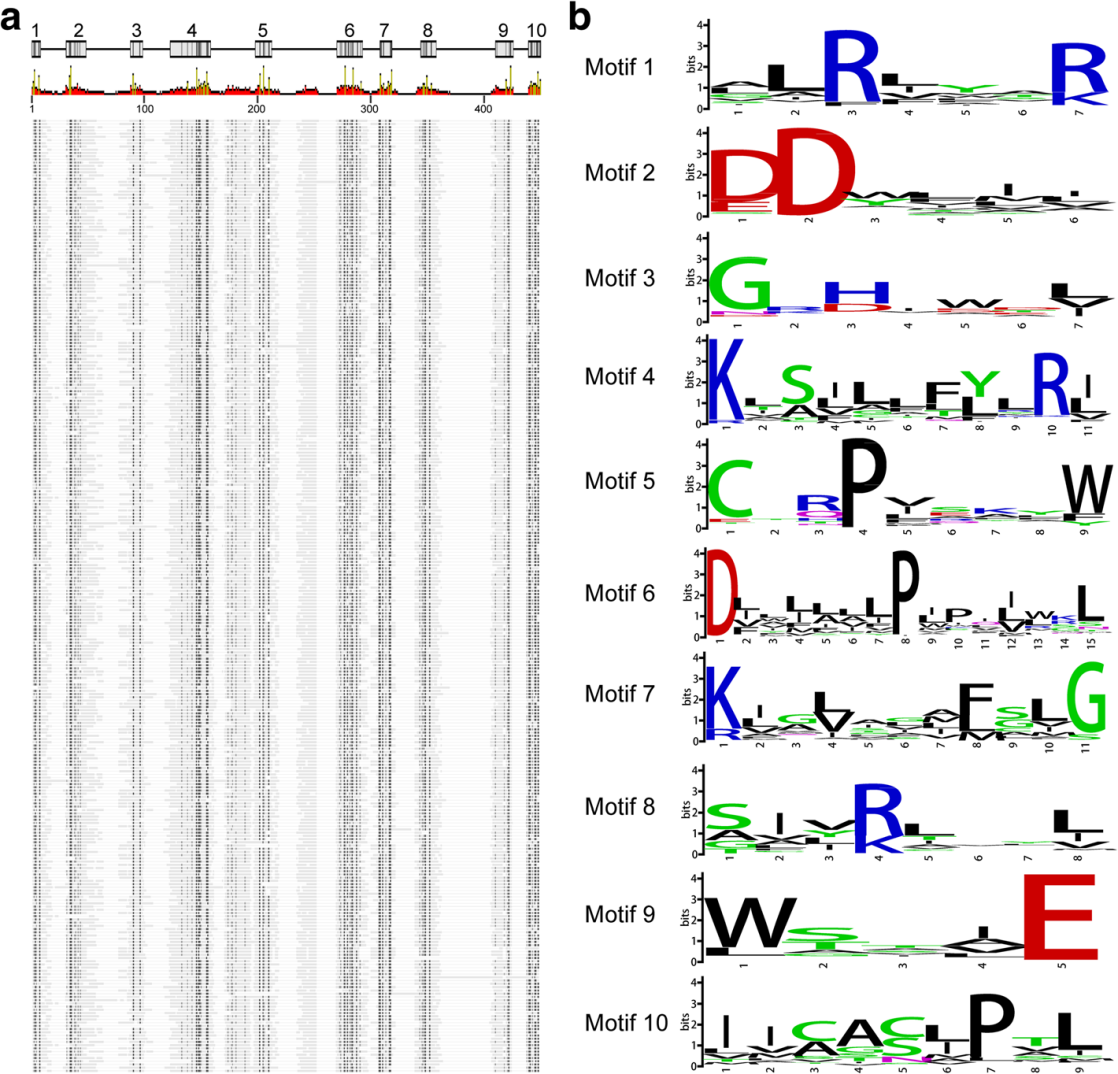
Multiple sequence alignments and conserved motifs in Pth11-domain. **a** Alignment of 296 putative Pth11-related GPCRs. Each line refers to one protein sequence. The high conserved residues were in black (*yellow in scale bar*) while the low conserved ones were in grey (*red in scale bar*). The ten conserved blocks were indicated above the scale bar. **b** Sequences of the ten motifs. The ten motifs were detected based on the sequence alignment and each motif respectively associated with a corresponding block

By multiple sequence alignments of all 296 putative Pth11-related GPCRs, ten blocks of conserved sequences in Pth11-domain were detected (Fig. 2a). Each block contained a conserved motif (Fig. 2b). For examples, motif 1, 2, and 3 were conserved in LXXXR, DD, and GXH/D patterns respectively. The conserved residues of Pth11-domain detected from the 20 proteomes were consistent with previously study in which proteins used were limited to *M. grisea* and *N. crassa* [25], indicating the high probability of authenticity of these putative Pth11-related GPCRs.

### Chromosomal distribution of putative Pth11-related GPCR genes

**Fig. 3:**
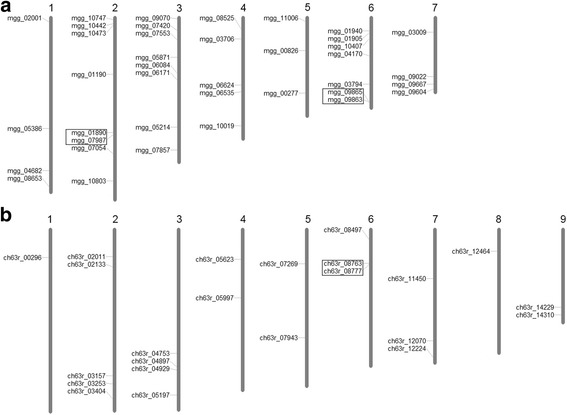
Chromosomal distribution of putative *M. oryzae* (**a**) and *C. higginsianum* (**b**) Pth11-related GPCR genes. The chromosome numbers are shown at the top of the chromosomes (*M. oryzae*) or scaffolds (*C. higginsianum*), and tandemly duplicated genes are shown in boxes. No putative Pth11-related GPCR genes were detected on scaffold 10 of *C. higginsianum*

To determine the chromosomal distribution of putative Pth11-related GPCRs, chromosome maps were constructed for *M. oryzae*, *C. higginsianum*, and *F. graminearum* (Fig. 3 and Additional file 1). These three species all have complete chromosomal (or scaffold) information available, encode more putative Pth11-related GPCRs than others, and belong to different orders. In *M. oryzae*, the putative Pth11-related GPCR genes are distributed among all seven chromosomes (Fig. 3a). Both chromosome 2 and 3 encoded the highest number (8 genes) of putative Pth11-related GPCR genes, followed by chromosome 6 and 4, encoding 6 and 5 genes respectively. Tandem duplications were found in the chromosome 2 and 6. In *C. higginsianum*, scaffold 10 was devoid of putative Pth11-related GPCR genes, whereas scaffold 2 encoded the maximum of 5 genes (Fig. 3b). Moreover, tandem duplication was found in scaffold 6. No tandem duplication of putative Pth11-related GPCR genes was found in *F. graminearum* (Additional file 1).

### Phylogenetic and hierarchical clustering analysis

**Fig. 4:**
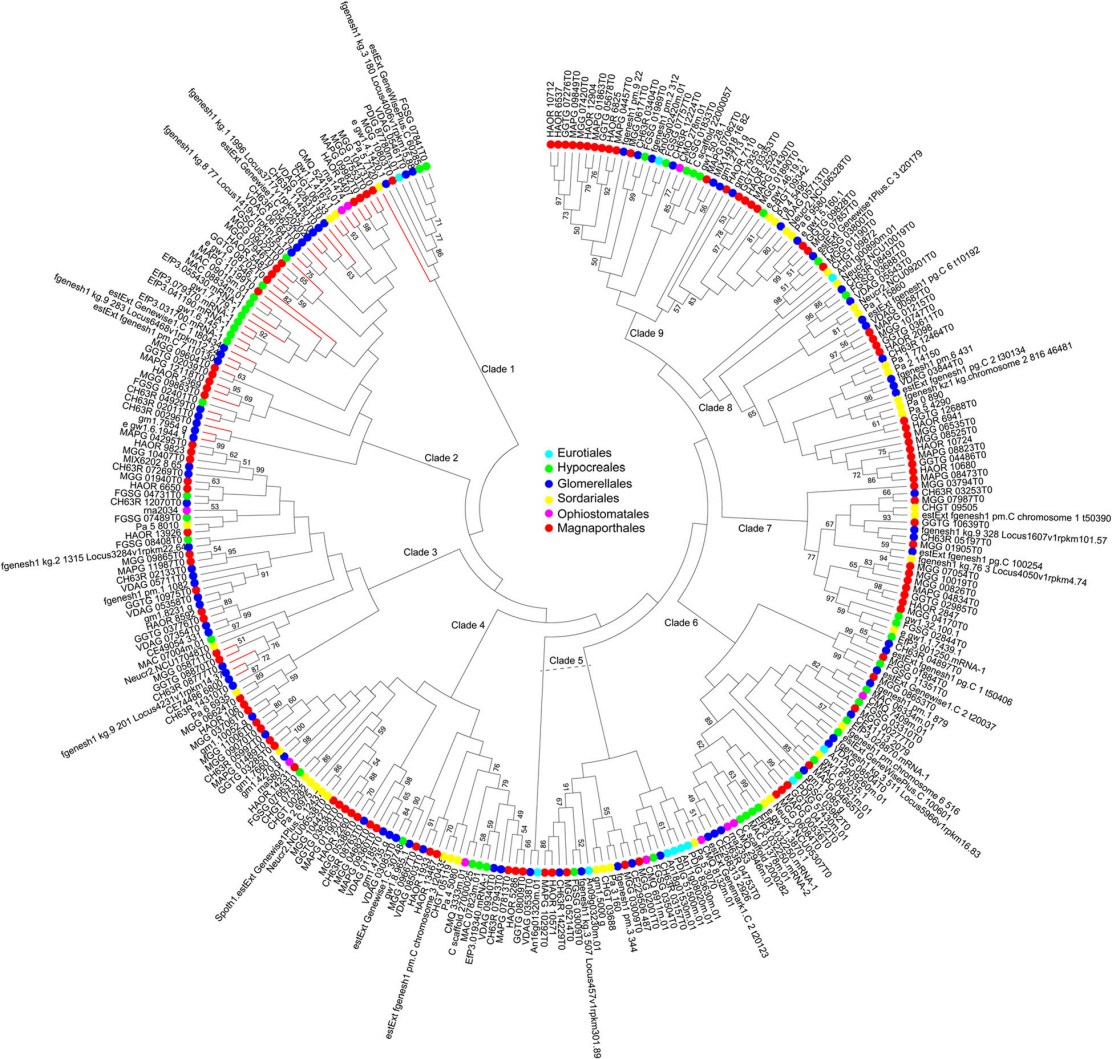
Maximum likelihood phylogenetic tree of all predicted Pth11-related GPCRs among 20 selected fungal species. Species belonging to Eurotiales, Hypocreales, Glomerellales, Sordariales, Ophiostomatales, and Magnaporthales are indicated by cyan, green, blue, yellow, purple, and red circles, respectively. The thick red lines denote Pth11-related GPCRs containing a CFEM domain. Bootstrap values greater than 50% are shown at branches

**Fig. 5:**
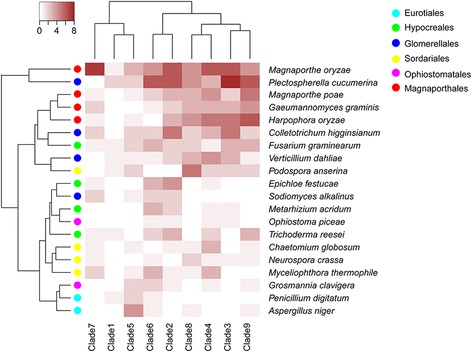
Hierarchical clustering analysis of 20 selected fungal species based on the counts of putative Pth11-related GPCRs in each clade. The clades are defined based on the phylogenetic tree in Fig. 4. Cyan, Eurotiales; Green, Hypocreales; Blue, Glomerellales; Yellow, Sordariales; Purple, Ophiostomatales; Red, Magnaporthales

To elucidate the evolutionary relationships among putative Pth11-related GPCRs across fungi in Pezizomycotina, a phylogenetic analysis was performed using the conserved regions of putative Pth11-related GPCR sequences (Fig. 4). Generally, the putative Pth11-related GPCRs from each taxonomic order (i.e., Magnaporthales, Ophiostomatales, Sordariales, Glomerellales, Eurotiales, and Hypocreales) were scattered throughout the phylogenetic tree rather than clustered together. According to the phylogenetic tree, all putative members of the Pth11-related GPCR family can be divided into nine major clades (Fig. 4). Almost all clades were comprised of putative Pth11-related GPCRs from different orders, and no order-specific clade was found. We also performed a hierarchical clustering analysis of the 20 species based on the counts of putative Pth11-related GPCRs in each clade (Fig. 5). This revealed that species with similar nutritional strategies from the same order clustered together, including Magnaporthales (*H. oryzae* arose from phytopathogens and can be considered a phytopathogen [27]), Sordariales, Eurotiales, and Glomerellales. Meanwhile, species from the same order, but with different nutritional strategies, did not cluster together. To be specific, the phytopathogen *F. graminearum* (Hypocreales) was clustered with phytopathogens instead of with other fungi in Hypocreales while the saprophyte *S. alkalinus* (Glomerellales) was clustered with saprophytes rather than other phytopathogens in Glomerellales. Entomopathogens shared a cluster with *E. festucae.* This may be because *E. festucae* was derived from insect-parasitic ancestors [28].

### CFEM domains in putative Pth11-related GPCRs

Only a subset of the identified putative Pth11-related GPCRs (46, 15%) contained cysteine-rich CFEM domain (Fig. 4 and Additional file 2). The *P. cucumerina* genome encodes the highest number of CFEM-containing Pth11-related GPCRs (8) followed by *M. oryzae* and *C. higginsianum* (6). All CFEM-containing Pth11-related GPCRs are included in two clades, i.e., clade 2 (25), and clade 3 (5), except for *P. anserina* Pa_5_7120 in clade 1, indicating that the sequences are very closely related. Similarly, Kulkarni et al. [25] showed that CFEM-containing Pth11-related GPCRs in *M. oryzae* and *N. crassa* occurred in one clade while Gruber et al. [29] showed that this type of GPCR in *T. reesei*, *T. atroviride*, and *T. virens* also clustered together. It is worth noticing that most of CFEM-containing Pth11-related GPCRs were detected in phytopathogens (67%).

### Expression patterns of putative Pth11-related GPCR genes in phytopathogens

**Fig. 6:**
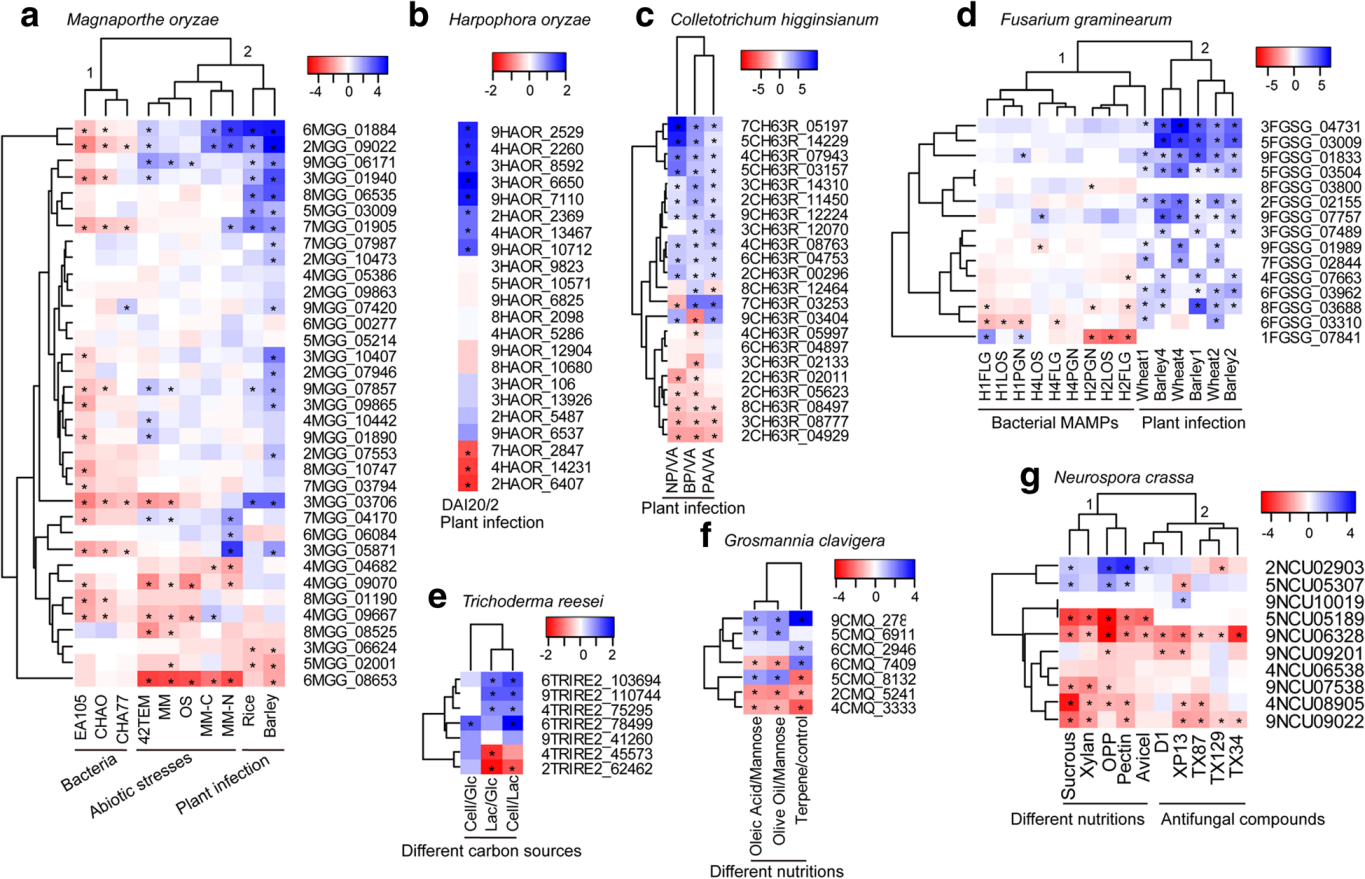
Heatmaps of gene expression of putative Pth11-related GPCRs. **a** Gene expression in *M. oryzae* under various treatments. EA105, CHAO, and CHA77 refer to bacteria, including a pseudomonad naturally isolated from rice soil, a *P. fluorescens* biocontrol strain, and the non-cyanide-producing mutant of CHAO, respectively. 42TEM: heat shock (42 °C for 45 min); MM, minimal media; OS, oxidative stress (treated with methyl viologen); MM-C, carbon limitation; MM-N, nitrogen limitation; Rice: rice at 72 h post-inoculation (hpi); Barley: barley at 72 hpi. **b** Comparative gene expression of *H. oryzae* between DAI20 and DAI2 which refer to genes expressed by *H. oryzae* infecting rice roots at 20 and 2 days after inoculation (DAI), respectively. **c** Comparative gene expression of *C. higginsianum* in four stages during the infection process of *Arabidopsis*. The four stages are: VA (in vitroappressoria), PA (in planta appressoria), BP (biotrophic phase), and NP (necrotrophic phase). **d** Gene expression of *F. graminearum* under various treatments. *F. graminearum* was treated with bacterial MAMPs including flagellin (FLG), lipooligosacharides (LOS), and peptidoglycans (PGN). Time points are 1, 2, and 4 h after treatment with MAMPs. Barley and wheat indicated gene expression during the infection time course (1, 2, and 4 days after inoculation). **e** Comparative gene expression of *T. reesei* growing on glucose (Glc), cellulose (Cell), or lactose (Lac) as a sole carbon source. **f** Comparative gene expression of *G. clavigera* growing on mannose, olive oil, oleic acid, or terpene as a sole carbon source. **g** Gene expression of *N. crassa* under various conditions. *N. crassa* was subjected to five different nutrient conditions [pectin, orange peel powder (OPP), xylan, avicel, or sucrose as a sole carbon source] and five antifungal compounds [three thioxanthone derivatives (TX129, TX34, TX87), XP13 (a prenylated analogue of 3,4-dihydroxyxanth-9-one,) and D1 (2,4-dihydroxy-3-methylacetophenone)]. Asterisks denote differential expressions greater than twofold change

In order to gain an insight into the possible function of Pth11-related GPCRs, we analyzed the expression patterns of putative Pth11-related GPCR genes under various conditions, including during biotic stress, invasive plant infection, and growth under different nutritional conditions (Fig. 6). Gene expression data for putative Pth11-related GPCRs was mined from publically available datasets, including experiments of GSE65311 [30] and GSE49597 [31] for biotic stress, GSE37886 [32], GSE21908 [33], and GSE33683 [34] for invasive plant infection, and GSE43006 [35], GSE53040 [36], GSE42692 [37], and GSE46155 [38] for nutritional stress. RNA-seq data for *H. oryzae* were also used to study the invasive infection of plants by *H. oryzae* [27].

Expressions of most putative Pth11-related GPCR genes were downregulated when *M. oryzae* was treated with bacteria that inhibit *M. oryzae* growth, including EA105 (a pseudomonad naturally isolated from rice soil), CHAO (a *Pseudomonas fluorescens* biocontrol strain) and CHA77 (the non-cyanide-producing mutant of CHAO) (Fig. 6a). Transcriptional expression was analyzed when *F. graminearum* was exposed to bacterial MAMPs (microbe-associated molecular patterns), such as flagellin (FLG), lipooligosacharides (LOS), and peptidoglycans (PGN) (Fig. 6d). Three time-points were used (1, 2, and 4 h after treatment with MAMPs). Similarly to the expression patterns in *M. oryzae*, most putative *F. graminearum* Pth11-related GPCR genes were downregulated during the biotic stress, especially after 2 h treatment (Fig. 6d). In contrast, upregulation of putative Pth11-related GPCR gene expression was detected during infection of both rice and barley by *M. oryzae*, including MGG_01884, MGG_09022, MGG_06171, MGG_06535, and MGG_01940 (Fig. 6a). The expressions of putative *F. graminearum* Pth11-related GPCRs during the infection time course 1, 2, and 4 days after inoculation of plants (wheat and barley) were compared to control (Fig. 6d). Consistent with *M. oryzae* again, most putative *F. graminearum* Pth11-related GPCR genes were upregulated during invasive plant infection. Besides, putative Pth11-related GPCR genes could also respond to invasive plant infection for *H. oryzae* (Fig. 6b) and *C. higginsianum* (Fig. 6c) by induced regulations with suppressions of a few of them. These results indicated that Pth11-related GPCRs can respond to both biotic stress and invasive plant infection, and in clearly different manners. The two different expression patterns were also suggested by the two separate clusters in the hierarchical clustering analysis (Fig. 6).

### Expression patterns of putative Pth11-related GPCR genes in saprophytes and entomopathogens

We examined putative Pth11-related GPCR gene expression in *N. crassa* treated with five antifungal compounds [three thioxanthone derivatives (TX129, TX34, TX87), XP13 (a prenylated analogue of 3,4-dihydroxyxanth-9-one), and D1 (2,4-dihydroxy-3-methylacetophenone)] and during growth under different nutritional conditions [media with either sucrose, xylan, pectin, orange peel powder (OPP), or avicel as a sole carbon source] (Fig. 6g). Almost all of the putative Pth11-related GPCRs were suppressed by the five antifungal compounds. Downregulation of putative Pth11-related GPCRs was also detected when *N. crassa* grew under given nutrient conditions. However, NCU02903 and NCU05307 were significantly induced by the 5 nutrient conditions. These two distinct expression patterns were supported by the fact that the GPCRs involved fell into two different clusters (Fig. 6g). Our results not only indicated that putative Pth11-related GPCRs of *N. crassa* can respond to different nutrient conditions and antifungal compounds, but also showed the clearly opposite expression manners responding to them. We also compared the gene expression of putative Pth11-related GPCR in *T. reesei* growing on glucose, cellulose, or lactose as carbon sources (Fig. 6e). Induced expression of most putative Pth11-related GPCRs was detected, indicating that a carbon source could affect the expression of putative *T. reesei* Pth11-related GPCR genes (Fig. 6e). Similar results were found for *G. clavigera*, which showed that a carbon source, including mannose, olive oil, oleic acid, and terpene, could alter the expression of putative *G. clavigera* putative genes (Fig. 6f).

## Discussion

It has been shown that Pth11 is involved in pathogenesis and is required for the plant pathogen *M. oryzae* to cause disease [25, 26]. Genomes of phytopathogens such as *P. cucumerina*, *V. dahlia* and *C. higginsianum* in Glomerellales and *M. oryzae*, *G.graminis*, and *M. poae* in Magnaporthales consistently have the largest number of putative Pth11-related GPCRs (from 16 to 41 genes). Compared with phytopathogens, much fewer putative Pth11-related GPCRs were detected in both saprophytes and entomopathogens. The phytopathogen *F. graminearum* encodes 17 putative pth11-related GPCRs, many more than the other three species (with 7 to 12 each) in Hypocreales. Both *H. oryzae* and *E. festucae* are symbionts. However, *H. oryzae* encodes much more pth11-related GPCRs than *E. festucae.* One possible explanation is that *E. festucae* is derived from insect-parasitic ancestors [28], while *H. oryzae* arose from phytopathogenic ancestors [27]. These results showed that the arsenal of Pth11-related GPCRs might be related to nutritional strategies, especially for phytopathogens. Besides, the hierarchical clustering analysis revealed that species with similar nutritional strategies from the same order clustered together while species from the same order, but with different nutritional strategies were detected in different clusters. These results revealed that instead of fungi from the same order, those fungi with similar nutritional strategies were inclined to share orthologs of putative Pth11-related GPCRs. Moreover, Pth11 has an extracellular amino-terminal CFEM domain [25, 39]. Although only a subset of putative Pth11-related GPCRs contained this fungal-specific cysteine-rich CFEM domain, most of CFEM-containing Pth11-related GPCRs were detected in phytopathogens and were very closely related. This phenomenon also indicated that trophic variations have been important factors in the evolution of Pth11-related GPCRs. Furthermore, the topology of the phylogenetic tree indicated that each order’s putative Pth11-related GPCRs were derived from GPCRs of their common ancestors and that Pth11-related GPCRs gained and/or lost several times during the evolutionary process. Overall, these results make it fairly safe to infer that Pth11-related GPCRs existed before the divergence of Pezizomycotina, and later evolved independently in a species-specific manner. And during the evolution of Pth11-related GPCRs, the different nutritional strategies of these fungi could be an important evolutionary stress.

The evolution of Pth11-related GPCRs involved in trophic variations of fungi in Pezizomycotina could also be revealed by their possible functions. We analyzed the expression pattern of putative Pth11-related GPCR genes under various conditions, including during biotic stress, invasive plant infection, and growth under different nutritional conditions. Expressions of most putative Pth11-related GPCR genes from both *M. oryzae* and *F. graminearum* were downregulated during the biotic stress while upregulation were detected during invasive plant infection by both of them. The two clearly different expression patterns revealed that Pth11-related GPCRs can respond to both biotic stress and invasive plant infection for phytopathogens. For saprophytes, almost all the putative Pth11-related GPCRs from *N. crassa* were suppressed by the antifungal compounds but some of them were induced when *N. crassa* were subjected to different nutrient conditions. Similar results were also found by Cabrera et al. [9], who revealed that many Pth11-related GPCRs are related to chemical sensitivity or nutritional phenotypes by analyzing the phenotypes of mutants. We also found that carbon source could affect the expression of putative Pth11-related GPCR genes of *T. reesei* and *G. clavigera*. The results revealed the common functions of Pth11-related GPCRs in respond to nutritional conditions for saprophytes and entomopathogens.

Expression patterns of putative Pth11-related GPCR genes in *M. oryzae* were also detected under abiotic stresses including growth on minimal medium, carbon and nitrogen starvation, heat shock (42 °C for 45 min), and oxidative stress (treated with methyl viologen). Interestingly, the hierarchical clustering analysis showed that the abiotic stress and invasive plant infection clustered together, indicating the similarity in expression patterns of putative Pth11-related GPCR genes between abiotic stress and invasive plant infection. These similar expression patterns may have resulted from that *M. oryzae* typically encountering nutrient-limited environments at the invasive growth stage [33].

The key role of tandem duplication in the evolution of other gene families has been reported, including the P450 family [40]. It has also been shown that gene duplication often plays a central role in both fungal and plant diversification, and is a key process generating the raw material necessary for adaptive evolution [41, 42]. Tandem duplications were detected in the chromosomes of both *M. oryzae* and *C. higginsianum*, indicating that tandem duplication may contribute to the evolution of the Pth11-related GPCR family. However, no tandem duplication of putative Pth11-related GPCR genes was found in *F. graminearum* (Additional file 1), indicating the complex evolutionary history of Pth11-related GPCRs.

## Conclusions

This study provided a thorough examination of 20 genomes in Pezizomycotina for putative Pth11-related GPCRs. The 20 selected fungi cover different nutritional strategies, including phytopathogens, symbionts, saprophytes, and entomopathogens. To elucidate the evolution of putative Pth11-related GPCRs, we performed a chromosome distribution and phylogenetic analysis of them. This analysis indicated that putative Pth11-related GPCRs existed before the divergence of Pezizomycotina, and that the GPCRs in each species were derived from GPCRs of their common ancestors. During this evolutionary process, putative Pth11-related GPCRs could have been gained and lost several times, possibly involving tandem duplication. Our results showed that putative Pth11-related GPCRs could respond to bacterial challenges, antifungal compounds, different nutritional conditions, and invasive plant infection, and different expression patterns were used to in response to these stimuli. Based on the common functions of putative Pth11-related GPCRs in respond to nutritional conditions and the results of fungi with similar nutritional strategies were inclined to share orthologs of putative Pth11-related GPCRs, we suggested that the different nutritional strategies of fungi could have been an important evolutionary stress during the evolution of Pth11-related GPCRs. It is worth mentioning that the proteins identified as putative Pth11-related GPCRs in this study have only been characterized in silico. Compared with only three types of G protein in most fungi, a large number of putative Pth11-related GPCRs were detected. Therefore, determining the intracellular interactions of Pth11-related GPCRs and their signaling output will help us understand how fungi adapt to different challenges and nutritional conditions.

## Methods

### Identification of putative Pth11-related GPCRs

Twenty genome sequences and deduced proteomes of Pezizomycotina were used from the following fungi: *M. oryzae*, *G. graminis*, *M. poae* [43], *H. oryzae* [44], *G. clavigera* [45], *C. globosum* [46], *N. crassa* [47], *V. dahlia* [48], *C. higginsianum* [49], *F. graminearum* [50], *E. festucae* [51], *M. acridum* [52], *T. reesei* [53], *P. digitatum* [54], *A. niger* [55], *S. alkalinus* [56], *P. cucumerina* [57], *P. anserine* [58], *M. thermophile* [59], and *O. piceae* [60]. Genome sequence of *S. cerevisiae* [61] was used as outgroup. These species cover Magnaporthales, Ophiostomatales, Sordariales, Glomerellales, and Hypocreales, and comprise phytopathogens (*M. oryzae*, *G. graminis*, *M. poae*, *V. dahlia*, *C. higginsianum*, *F. graminearum* and *P. cucumerina*), symbionts (*H. oryzae*, and *E. festucae*), saprophytes (*O. piceae, N. crassa, C. globosum, M. thermophile, P. anserina, S. alkalinus, T. reesei, A. niger, and P. digitatum*), and entomopathogens (*G. clavigera* and *M. acridum*). A pipeline was used to identify the putative Pth11-related GPCRs in the 21 selected proteomes. Firstly, as the Pth11-domain distinguishes Pth11-related proteins from other class of GPCR-like proteins [25], the hmmscan program from the HMMER v3.1 package [62] was used to search for the Pth11-domain across all the 21 proteomes with an e-value cutoff of 1e-20. Then the obtained Pth11-domain containing proteins were used in a BLASTP search against Pth11-related GPCRs of *M. oryzae* [25]. An e-value limit of 1e-09 was applied, and all proteins that had at least 30% identity and 80% overlap over the length of the proteins were retained [25]. Finally, the obtained proteins were evaluated for the typical seven-transmembrane domain by TMHMM, HMMTOP, and Phobius [63–65], and proteins with seven or more transmembrane domains predicted by at least two algorithms were retained as putative Pth11-related GPCRs and used for further analysis. By this analysis pipeline, the authenticity of these predicted Pth11-related GPCRs was highly improved.

### Chromosomal organization of putative Pth11-related GPCRs

Chromosome location images were generated using MapInspect software to localize putative Pht11-related GPCRs of *M. oryzae*, *C. higginsianum*, and *F. graminearum*. Any putative Pth11-related GPCRs separated by no more than 15 genes were identified as tandemly duplicated genes.

### Protein alignments and phylogenetic analysis

Protein sequences were aligned using ClustalW v2 [66]. To select conserved regions, the alignments were analyzed with Gblocks v0.91b [67] using the default parameters. The best amino acid substitution model was chosen using ProtTest v3.2 [68], and LG + G + F model was selected as the best-fit model for the datasets. The phylogenetic tree of putative Pth11-related GPCRs was constructed in MEGA v7 [69] using maximum likelihood (ML) methods with the best-fit model followed by bootstrap analysis (1000 bootstrap replications). To infer phylogenetic relationships among the 20 species, 100 clusters of 1:1 orthologs were chosen based on our previous study [27]. The proteins of the 100 orthologs were aligned and then concatenated. Phylogenetic analyses were performed using the ML criterion implemented in RAxML [70] through the RAxML-HPC BlackBox web server at the Cyber Infrastructure for Phylogenetic Research with LG + G + I model. The sequence logo was created using WebLogo v2.8.2 [71] based on a multiple sequence alignment of 296 putative Pth11-related GPCRs.

### CFEM domain search

The CFEM domain architectures of putative Pth11-related GPCRs were predicted using two search methods, including Pfam [72], and Conserved Domain Search (CDSearch) [73] applying the default settings for each.

### Expression analysis

Expression data were mined for putative Pth11-related GPCR genes from several datasets, including RNA sequencing and microarray data, which were downloaded from the Gene Expression Omnibus. The datasets with accession numbers GSE65311, GSE37886 (*F. graminearum*), GSE21908, GSE49597 (*M. oryzae*), GSE33683 (*C. higginsianum*), GSE43006 (*G. clavigera*), GSE53040, GSE42692 (*N. crassa*), and GSE46155 (*T. reesei*) were analyzed. For *H. oryzae*, RNA-seq data from a previous study [27] were used. These data pertained to different environmental conditions, including biotic and abiotic stress conditions and various stages of colony development. The data were visualized using heatmaps generated with the heatmap.2 package in R, which is based on log2 fold changes after normalization.

## Additional files

Additional file 1: Chromosomal distribution of putative *F. graminearum* Pth11-related GPCR genes. Chromosome numbers are shown at the top of the chromosomes. (TIFF 39 kb)

Additional file 2: Predicted Pth11-related GPCRs among thirteen fungi belonging to Pezizomycotina. (XLSX 24 kb)

## Abbreviations

BLAST: Basic local alignment search tool
GPCR: G-protein coupled receptors
GPCRDB: GPCR database
MAMP: Microbe-associated molecular patterns
