# Supplementary figures and images for: Genome-wide comparative analysis of putative Pth11-related G protein-coupled receptors in fungi belonging to Pezizomycotina
Source: BMC Microbiol. 2017 Jul 25;17:166. doi: 10.1186/s12866-017-1076-5 (PMC5526305; doi:10.1186/s12866-017-1076-5)

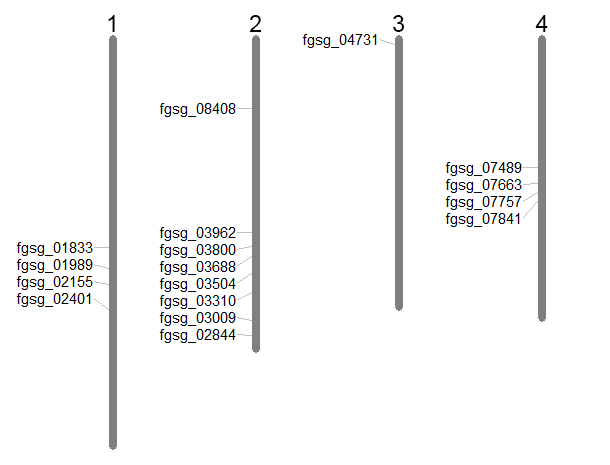

Supplement: Supplementary file 1 — Chromosomal distribution of putative F. graminearum Pth11-related GPCR genes. Chromosome numbers are shown at the top of the chromosomes. (TIFF 39 kb) [file 12866_2017_1076_MOESM1_ESM.tif]
